# Left Bundle Branch Area Pacing vs. Biventricular Pacing for Cardiac Resynchronization: Propensity Score Analysis

**DOI:** 10.1002/joa3.70359

**Published:** 2026-05-06

**Authors:** Fawzi Kerkouri, Pierre Khattar, Vincent Mansourati, Laurent Palud, Jean Baptiste Moreau, Hugo Hager, Jean Philippe Hacot, Florent Le Ven, Jacques Mansourati

**Affiliations:** ^1^ Department of Cardiology University Hospital of Brest Brest France; ^2^ Univ Brest, Laboratoire ORPHY EA 4324 Brest France; ^3^ Department of Cardiology Hospital of Lorient Lorient France

**Keywords:** arrythmias, conduction system pacing, CRT, heart failure, left ventricular ejection fraction

## Abstract

**Background:**

Left bundle branch area pacing (LBBAP) has emerged as a promising alternative to conventional biventricular pacing (BIVP) for cardiac resynchronization therapy (CRT). While previous data suggest LBBAP may provide superior outcomes, existing evidence needs more data.

**Methods:**

This retrospective study included all patients who underwent de‐novo CRT at two centers in France (2022–2024). Procedural and clinical outcomes were compared between LBBAP and BIVP groups for de‐novo CRT indications, using inverse probability weighting propensity score.

**Results:**

A total of 314 patients were included (75 LBBAP, 239 BIVP). Patients receiving LBBAP were older and more likely to have atrial fibrillation. Compared to BIVP, LBBAP was associated with shorter procedure time (96 vs. 128 min; *p* < 0.001), narrower paced QRS (128 vs. 139 ms; *p* < 0.001), and lower post‐discharge device‐related complications (1% vs. 11%; HR 0.10, 95% CI 0.01–0.84; *p* = 0.033). At 1‐year, improvement in left ventricular ejection fraction (LVEF) was similar between groups (median ΔLVEF +15%; *p* = 0.438), as were hyper‐response rates (52% vs. 60%; *p* = 0.231). After IPW‐PS, no significant differences were observed in heart failure hospitalization (HR 0.99, 95% CI 0.40–2.64; *p* = 0.999), new‐onset atrial fibrillation (HR 1.69, 95% CI 0.41–6.94; *p* = 0.492), sustained ventricular arrhythmias (HR 1.23, 95% CI 0.15–6.49; *p* = 0.828), overall (HR 1.10, 95% CI 0.44–2.62; *p* = 0.801), and cardiovascular deaths (HR 0.92, 95% CI 0.27–2.79; *p* = 0.927).

**Conclusion:**

LBBAP is associated with comparable resynchronization and clinical outcomes to BIVP, with fewer late complications.

## Graphical abstract

In this two‐center propensity score–weighted cohort of de novo CRT recipients, left bundle branch area pacing was associated with comparable resynchronization and clinical outcomes to biventricular pacing, with fewer late device‐related complications.

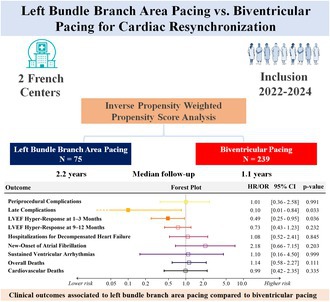

**Abbreviations**
AF: Atrial fibrillation
BIVP: Biventricular pacing
CRT: Cardiac resynchronization therapy
IPW‐PS: Inverse probability weighting propensity score
LBBAP: Left bundle branch area pacing
LBBB: Left bundle branch block
LV: Left ventricle / ventricular
LVEF: Left ventricular ejection fraction
NYHA: New York Heart Association

## 1 Introduction

Biventricular pacing (BIVP) is a guideline‐recommended therapy for patients with reduced left ventricular ejection fraction (LVEF) and electrical desynchrony—particularly left bundle branch block (LBBB) with QRS ≥ 150 ms [1]. Randomized trials show that near‐simultaneous activation of both ventricles improves cardiac pump function, promotes reverse remodeling, reduces heart failure hospitalizations, and improves survival [2, 3, 4]. Yet up to one‐third of candidates derive little or no clinical benefit (“non‐responders”). Major determinants include suboptimal lead position, myocardial scar (notably in ischemic heart disease), and residual asynchrony inherent to epicardial left ventricular (LV) pacing [5, 6]. Coronary sinus anatomy and phrenic nerve stimulation can also limit or preclude BIVP.

Conduction system pacing has emerged as a physiologic alternative. His‐bundle pacing (HBP) demonstrated feasibility but is constrained by high thresholds and lead instability [7, 8]. Left bundle branch area pacing (LBBAP), introduced in 2017, captures the distal His–Purkinje system with lower thresholds and more stable parameters, yielding narrow QRS complexes and favorable hemodynamics [9]. To date, evidence remains limited: one small randomized pilot and several modest observational studies report mixed findings—some suggesting greater LVEF and symptomatic gains with LBBAP versus BIVP [10, 11, 12, 13], others showing equivalent remodeling [14, 15]. Accordingly, consensus statements continue to position BIVP as first‐line cardiac resynchronization therapy (CRT), with LBBAP as an alternative [16].

Given the need for more evidence in this field, we compared real‐world clinical outcomes of LBBAP versus BIVP in patients undergoing de novo CRT for heart failure, using a rigorous methodology with propensity score analysis.

## 2 Methods

### 2.1 Study Design and Patient Population

We conducted a retrospective cohort study including consecutive patients who underwent de novo cardiac resynchronization therapy (CRT) with either biventricular pacing (BIVP) or left bundle branch area pacing (LBBAP) at two centers in Western France: Brest University Hospital and Lorient Hospital. The study period spanned from January 1, 2022, to December 31, 2024—a timeframe selected after operators at both centers had gained substantial experience with LBBAP (> 50 non‐CRT implants per operator). This ensured procedural maturity and comparable contemporary heart failure management. Eligible patients met guideline‐based indications for CRT, typically defined by LVEF ≤ 35%, New York Heart Association (NYHA) class II–III symptoms, and QRS prolongation, or anticipated high ventricular pacing burden in the context of LV dysfunction.

**FIGURE 1:**
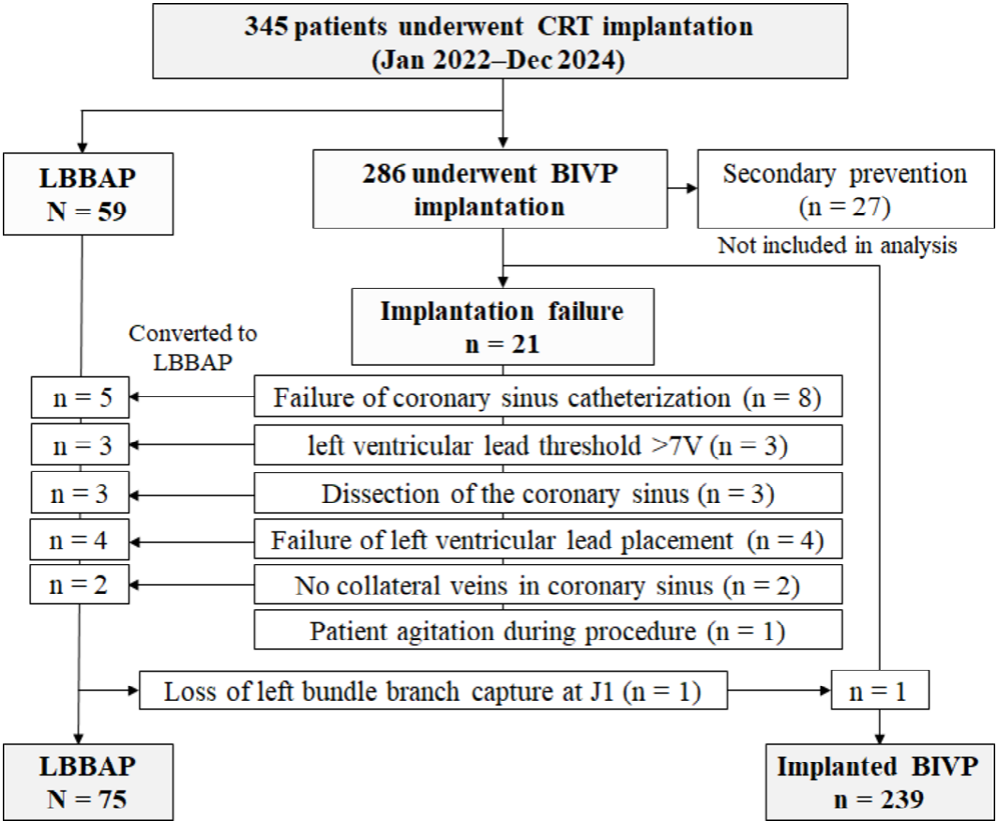
Study flow of BIVP and LBBAP implantations. Patients were analyzed according to the final implanted pacing strategy (as‐treated analysis). CRT denotes cardiac resynchronization therapy, BIVP denotes biventricular pacing, and LBBAP denotes left bundle branch area pacing.

Patients were categorized according to the implant strategy. The BIVP group comprised those who underwent conventional CRT implantation with right ventricular and coronary sinus leads, whereas the LBBAP group included patients receiving pacing at the left bundle branch area, either as a primary approach or as a rescue strategy after unsuccessful coronary sinus lead placement (Figure 1). The choice between CRT‐pacemaker (CRT‐P) and CRT‐defibrillator (CRT‐D) systems was made by the treating electrophysiologist and heart failure team. Patients implanted for secondary prevention (i.e., prior sustained ventricular arrhythmia or aborted cardiac arrest requiring defibrillator therapy) were excluded to minimize baseline heterogeneity, as all such cases underwent BIVP‐CRT‐D implantation. The primary analysis was performed according to the final implanted pacing strategy (as‐treated analysis), and crossover cases were classified according to the device ultimately implanted and functioning after the index procedure.

### 2.2 Data Collection

Baseline clinical, procedural, and follow‐up data—including all‐cause and cause‐specific mortality—were retrospectively extracted from electronic medical records between August 15 and October 23, 2025. Standardized definitions were applied to ensure consistency and minimize reporting bias see (Tables S1 and S2). Follow‐up duration was calculated from the date of device implantation to the date of last clinic contact or death; patients alive at last follow‐up were censored at that date.

In both centers, routine device clinic visits were scheduled at 1–3 months and 9–12 months post‐implantation. The same surveillance protocol was applied in both groups throughout follow‐up. At these follow‐up visits, transthoracic echocardiography was performed in conjunction with device interrogation to confirm effective resynchronization and optimize device programming when clinically indicated. When pacing percentage was suboptimal, the underlying cause was systematically investigated and corrected whenever possible. Serial assessment of LVEF was based exclusively on transthoracic echocardiography, and changes in LVEF between implantation and follow‐up were derived from echocardiographic measurements. To minimize loss to follow‐up, referring cardiologists or physicians were systematically contacted to verify outcomes. Vital status was verified through the National Institute of Statistics and Economic Studies (INSEE) national registry, which records all deaths in France. Causes of death were classified according to European Society of Cardiology (ESC) guidelines as cardiovascular, non‐cardiovascular, or unknown [17]. Cardiovascular death encompassed all deaths due to cardiac or vascular causes. Sudden cardiac death (SCD) was defined as a sudden, natural death of presumed cardiac origin occurring within 1 h of symptom onset (witnessed) or within 24 h of last being seen alive (unwitnessed). Sudden unexplained deaths presumed non‐cardiac were categorized as unknown.

All outcomes were adjudicated blinded to pacing strategy. Data management and statistical analyses were performed by the Clinical Investigation Center (INSERM U1412), Brest University Hospital.

### 2.3 Endpoints

The prespecified endpoints for comparing LBBAP vs. BIVP were as follows: (1) Peri‐procedural major complications: any device‐ or procedure‐related adverse event occurring from implantation to hospital discharge that resulted in death, required invasive reintervention, or prolonged hospitalization by > 48 h. (2) Late major complications: device‐related complications occurring after hospital discharge that resulted in death, required invasive reintervention, or led to hospital readmission. (3) LVEF response: assessed at early (1–3 months) and late (9–12 months) follow‐up. The absolute change from baseline was calculated at each time point. Patients were classified as hyper‐responders (≥ 15‐point increase), partial responders (5–< 15‐point increase), or non‐responders (< 5‐point increase or any decline). Mean LVEF at each time point was also compared between groups. (4) Hospitalizations for decompensated heart failure. (5) New‐onset atrial fibrillation (AF): assessed only in patients without prior AF at baseline and defined as the first documented episode of AF during follow‐up, lasting > 30 s on a standard electrocardiogram or on 24‐ to 72‐h Holter monitoring. (6) Sustained Ventricular Arrhythmias. (7) Mortality (Overall and Cardiovascular).

### 2.4 Statistical Analysis

Baseline characteristics were summarized as counts (percentages), means (standard deviation), or medians [interquartile range], as appropriate. Group comparisons were performed using appropriate statistical tests. Outcomes were expressed as incidence rates per 100 person‐years, and risk differences quantified the time‐adjusted absolute effect of pacing modality.

Associations between CRT type and outcomes were assessed using logistic regression for binary endpoints (periprocedural major complications, early and late LVEF hyper‐response), linear regression for changes in LVEF (ΔLVEF at 30–90 days and at 9–12 months), and Cox proportional hazards models for time‐to‐event outcomes, including late major complications, hospitalization for decompensated heart failure, new‐onset AF, sustained ventricular arrhythmias, and mortality. All patients were included in endpoint analyses except for the analysis of new‐onset AF, which was restricted to patients without a prior history of AF at baseline. Standard errors were adjusted for clustering by operator.

Three approaches were utilized for comparison (1) crude models providing unadjusted hazard rations (HRs) or odds ratios (ORs) (2) multivariable models adjusted for clinically relevant covariates associated with each outcome (Table S3); and (3) inverse probability weighting based on the propensity score (IPW‐PS), estimated by logistic regression including baseline and outcome‐related covariates, including age, sex, body mass index, heart disease subtype, rhythm at implantation, QRS morphology, QRS duration, baseline LVEF, late gadolinium enhancement, right ventricular dysfunction, NYHA class, AF status/subtype, dialysis or severe chronic kidney disease, severe respiratory disease, psychiatric disorder/dementia, cirrhosis, cancer/malignant hematologic disease, impaired mobility, transcatheter aortic valve implantation, prior pacemaker, heart failure therapies, and anticoagulant use. IPW was preferred over propensity score matching to retain the entire cohort, and adequate covariate balance (standardized mean difference < 0.10) was confirmed using Love plots (Figure S1). Variables showing residual imbalance (SMD > 0.10) after weighting were subsequently included as additional covariates in the weighted outcome models to further reduce residual confounding. Because follow‐up duration is a post‐implant variable, differences in follow‐up time between groups were not included in the propensity score model. Instead, unequal follow‐up time was addressed in the time‐to‐event analyses using Cox proportional hazards models with time from implantation as the time scale and censoring at device removal, loss to follow‐up, or end of study.

Missing data were handled using multiple imputation. The proportional hazards assumption was verified using Schoenfeld residuals. A two‐tailed *p* value < 0.05 was considered statistically significant. All analyses were performed using R software, version 4.4.1.

## 3 Results

### 3.1 Patients Characteristics and Peri‐Procedural Outcomes

During the 3‐year study period, 287 patients underwent attempted BIVP implantation and 59 underwent LBBAP as the initial strategy. Among BIVP attempts, 21 patients (7.3%) experienced failure to place a functional LV lead; in 17 of these cases (81.0%), operators successfully implanted an LBBAP lead during the same procedure, accounting for 22.7% of all LBBAP implants. One patient in the LBBAP group was switched to BIVP on day 1 post‐implant because of loss of conduction capture, resulting in final cohorts of 75 LBBAP and 239 BIVP patients (Figure 1).

Baseline characteristics were broadly comparable between groups with respect to underlying heart disease, LVEF, LV dimensions, QRS morphology, NYHA class, comorbidities (hypertension, diabetes, hypercholesterolemia, AF, respiratory disease, cancer), and background medical therapy (beta‐blockers, aldosterone antagonists, loop diuretics, anticoagulants) (Table 1). Patients in the LBBAP group were older (75.1 ± 10.0 vs. 70.5 ± 10.1 years; *p* < 0.001), more likely to have chronic AF (41.3% vs. 24.7%; *p* < 0.001), advanced renal disease (21.3% vs. 10.9%; *p* = 0.020), and pacemaker history (2.5% vs. 16.0%; *p* < 0.001), and were less often treated with sacubitril/valsartan (61.3% vs. 75.3%; *p* = 0.019) or SGLT2 inhibitors (64.0% vs. 84.1%; *p* < 0.001).

**TABLE 1:** Baseline Characteristics for patients undergoing LBBAP. versus BIVP.

Characteristic^a^	LBBAP. (*n* = 75)	BIVP. (*n* = 239)	*p*
Age—years	75.1 ± 10.0	70.5 ± 10.1	< 0.001
Female Sex	28 (37.3%)	66 (27.6%)	0.109
Body mass index—kg/m^2^	25.9 [22.4–28.4]	26.2 [23.5–29.1]	0.142
**Rhythm at implant**			0.076
Sinus rhythm	49 (65.3%)	181 (75.7%)	
‐ PR interval > 200 ms	10 (20.4%)	48 (26.8%)	0.361
Atrial fibrillation	26 (34.7%)	58 (24.3%)	
**LBBB**	60 (80.0%)	201 (84.1%)	0.408
Typic LBBB	53 (88.3%)	193 (96.0%)	0.050
QRS duration—ms	156 [145–160]	160 [149–168]	0.155
**Heart disease**			0.664
Dilated cardiomyopathy	48 (64.0%)	139 (58.2%)	
Ischemic heart disease	20 (26.7%)	73 (30.5%)	
Other	7 (9.33%)	27 (11.3%)	
NYHA class	2.2 ± 0.5	2.2 ± 0.4	0.760
LVEF—%	30 [25–35] 29.8 ± 7.2	30 [25–33] 28.2 ± 6.5	0.071
LVEDV^b^—ml/m^2^	108 [99–117]	112 [96–128]	0.090
RV dysfunction	0 (0%)	10 (4.2%)	0.125
**Cardiac MRI performed**	13 (17.3%)	63 (26.4%)	0.111
Late Gadolinium Enhancement	5 (38.5%)	36 (57.1%)	0.219
Number of segments	4.2 ± 3.5	3.2 ± 2.6	0.169
Hypertension	42 (56.0%)	134 (56.1%)	0.992
Diabetes	21 (28.0%)	72 (30.1%)	0.725
Hypercholesterolemia	38 (50.7%)	109 (45.6%)	0.444
Coronary Artery Disease	34 (45.3%)	115 (48.1%)	0.674
**Atrial Fibrillation**	44 (58.7%)	130 (54.4%)	0.516
Burden			0.044
‐ Paroxysmal	13 (29.5%)	61 (46.9%)	
‐ Persistent/Permanent	31 (70.5%)	69 (53.1%)	
Severe respiratory disease	6 (8.0%)	23 (9.6%)	0.672
Significant psychiatric troubles/Dementia	3 (4.0%)	18 (7.5%)	0.285
Dialysis/Severe CKD	16 (21.3%)	26 (10.9%)	0.020
Cirrhosis	0 (0%)	8 (3.4%)	0.206
**Cancer/Malignant hematology**	17 (22.7%)	55 (23.0%)	0.950
Status			0.132
‐ Active	9 (52.9%)	18 (32.7%)	
‐ In Remission	8 (47.1%)	37 (67.3%)	
Impaired mobility	14 (18.7%)	26 (10.9%)	0.078
Transaortic valve replacement	4 (5.3%)	26 (10.9%)	0.154
History of Pacemaker	6 (2.5%)	12 (16.0%)	< 0.001
Valsartan/Neprilysin	46 (61.3%)	180 (75.3%)	0.019
Beta‐blocker	65 (86.7%)	202 (84.5%)	0.649
SGLT2 inhibitor	48 (64.0%)	201 (84.1%)	< 0.001
Aldosterone antagonist	37 (49.3%)	129 (54.0%)	0.482
Furosemide	40 (53.3%)	122 (51.0%)	0.729
ACEi/ARB	19 (25.3%)	30 (12.6%)	0.008
Anticoagulant	50 (66.7%)	137 (57.3%)	0.150

^a^ Data are mean ± sd, median [IQR], or *n* (%) as appropriate.

^b^ Missing data: 11 for LBBA Pacing, 30 for CRT. LBBAP denotes left bundle branch area pacing, BIVP cardiac resynchronization therapy with biventricular pacing, NYHA New York Heart Association functional class, LVEF left ventricular ejection fraction, LVEDV left ventricular end‐diastolic volume, and LBBB left bundle branch block.

Procedural efficiency favored LBBAP, with shorter skin‐to‐skin time (96 min [58–121] vs. 128 min [100–157]; *p* < 0.001) and reduced fluoroscopy exposure (18 min [12–34] vs. 23 min [16–37]; *p* = 0.018). The proportion of CRT‐D implants was lower in the LBBAP group (29.3% vs. 55.2%; *p* < 0.001), reflecting the older and more comorbid population. Electrophysiologic performance also favored LBBAP, with shorter paced QRS duration (128 ms [119–139] vs. 139 ms [127–148]; *p* < 0.001). Paced QRS morphology in lead V1 less frequently showed a right ventricular pre‐excitation pattern with LBBAP than with BIVP (86.7% vs. 94.1%; *p* = 0.034). Median stimulus‐to‐LV activation time (LVAT) was 75 ms [69–85], and the V1–V6 inter‐peak interval was 40 ms [35–45]. Additional procedural data are provided in Table S4.

Major periprocedural complications occurred at similar rates in both groups (LBBAP 10.7% vs. BIVP 8.4%; *p* = 0.54) (Table 2, Tables S5 and S6). The most frequent events were pocket hematoma and pneumothorax. One LBBAP patient died from a hemothorax complicated by infection. Predischarge reoperations were also comparable (6.7% vs. 3.8%; *p* = 0.290; Table S7). After inverse probability weighting, LBBAP remained associated with a similar risk of acute complications (adjusted OR 1.01; 95% CI 0.36–2.58; *p* = 0.991).

**TABLE 2:** Early and Late Major Complications.

Characteristic^a^	LBBAP. (*n* = 75)	BIVP (*n* = 239)	*p*
Pre‐discharge major complications	8 (10.7%)	20 (8.4%)	0.542
Pocket hematoma	2 (2.7%)	9 (3.8%)	> 0.999
Pericardial effusion requiring drainage	0 (0%)	1 (0.4%)	> 0.999
Lead macro‐dislodgement	0 (0%)	4 (1.7%)	0.576
Loss of LBB capture requiring reoperation	1 (1.3%)	0 (0%)	0.239
Pneumothorax	2 (2.7%)	3 (1.3%)	0.596
Coronary sinus dissection	0 (0%)	2 (0.8%)	> 0.999
Death related to implantation	1 (1.3%)	0 (0%)	0.239
Other^b^	2 (2.7%)	1 (0.4%)	0.143
Late major complications	1 (1.3%)	27 (11.3%)	0.008
Hematoma	0 (0%)	2 (0.8%)	> 0.999
Infection	0 (0%)	6 (2.5%)	0.342
Lead dislodgment	0 (0%)	16 (6.7%)	0.016
Loss of LBB capture requiring reoperation	1 (1.3%)	0 (0%)	0.239
Other	0 (0%)	5 (2.1%)	0.343

^a^ Data are *n* (%).

^b^ Details in Table S5.

### 3.2 Follow Up and Clinical Outcomes According to CRT Modality

Median follow‐up duration was 1.1 years [0.4–1.7] in the LBBAP group and 2.2 years [1.2–2.9] in the BIVP group (*p* < 0.001). In crude analysis, LBBAP was associated with a significantly lower rate of device‐related late complications (1.1 vs. 5.6 events per 100 person‐years; HR 0.11, 95% CI 0.01–0.51; *p* = 0.029) (Table 3). The only late event in the LBBAP group was a loss of capture due to micro‐dislodgement, successfully corrected by lead revision. In contrast, BIVP complications were predominantly LV lead–related, including dislodgment (6.7%), phrenic nerve stimulation (1.7%), and infection (2.5%). Late reoperation rates were also lower with LBBAP (1.1 vs. 4.0 events per 100 person‐years; *p* = 0.040). After adjustment using inverse probability weighting based on the propensity score, late complications remained significantly lower with LBBAP (HR 0.10, 95% CI 0.01–0.84; *p* = 0.033) (Figure 3).

**TABLE 3:** Outcomes Standardized by Follow‐Up Duration.

Characteristic^a^	LBBAP (*n* = 75)	BIVP (*n* = 239)	Rate/100 py (LBBAP vs BIVP)	Rate difference (/100 py)	*p*
Follow‐up duration—years	1.1 [0.4–1.7]	2.2 [1.2–2.9]	—	—	< 0.001
LVEF at 1–3 months	39.9 ± 9.0 40 [34–46]	41.3 ± 10.5 40 [35–50]	—	—	0.459
ΔLVEF at 1–3 months	10.1 ± 8.8	13.1 ± 10.8			0.022
hyper‐response at 1–3 months	29 (38.7%)	121 (50.6%)	—	—	0.070
Partial‐response at 1–3 months	27 (36.0%)	64 (26.8%)	—	—	0.141
Non‐response at 1–3 months	19 (25.3%)	54 (22.6%)	—	—	0.655
LVEF at 9–12 months	42.4 ± 11.5 45 [35–50]	44.3 ± 12.1 45.0 [35–52]	—	—	0.438
ΔLVEF at 9–12 months	12.6 ± 12.0	16.1 ± 12.1			0.040
hyper‐response at 9–12 months	39 (52.0%)	143 (59.8%)	—	—	0.231
Partial‐response at 9–12 months	20 (26.7%)	60 (25.1%)	—	—	0.884
Non‐response at 9–12 months	16 (21.3%)	36 (15.1%)	—	—	0.240
Late major complications	1 (1.3%)	27 (11.3%)	1.1 vs. 5.6	−4.5	0.030
Late reoperations	1 (1.3%)	19 (7.9%)	1.1 vs. 4.0	−2.9	0.040
Hospitalization for HF	10 (13.3%)	34 (14.2%)	11.1 vs. 7.1	+4.0	0.390
New onset of AF	4 (5.3%)	12 (5.0%)	4.9 vs. 2.3	+2.6	0.920
Sustained ventricular arrythmias	2 (2.7%)	13 (5.4%)	2.4 vs. 2.5	−0.1	0.740
Overall deaths	13 (17.3%)	25 (10.5%)	14.4 vs. 5.2	+9.2	0.070
Cardiovascular	5 (6.7%)	9 (3.8%)	5.6 vs. 1.9	+3.7	0.110
Non‐cardiovascular	5 (6.7%)	9 (3.8%)	5.6 vs. 1.9	+3.7	0.110

^a^ Data are mean ± sd, median [IQR], or *n* (%) as appropriate.

**FIGURE 2:**
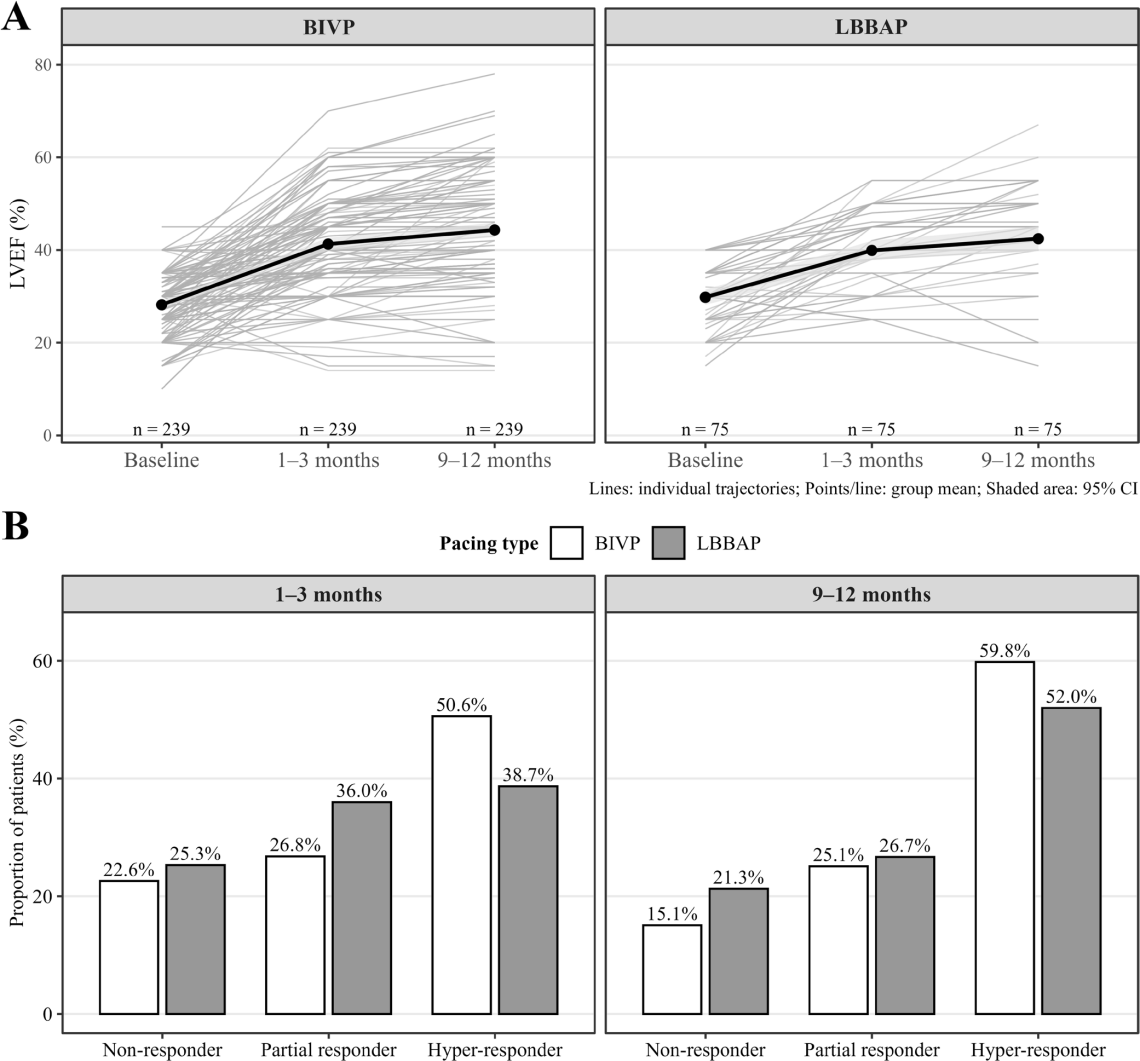
(A) Evolution of LVEF over time; (B) Response to BIVP or LBBAP over time. BIVP denotes biventricular pacing; LBBAP, left bundle branch area pacing. Responders were classified as partial (LVEF increase < 15%) or hyper‐responders (LVEF increase ≥ 15%); non‐responders showed no significant improvement in LVEF.

LVEF improved significantly in both groups from baseline to 9–12 months (Figure 2A). In linear regression, patients receiving LBBAP exhibited a modestly smaller early ΔLVEF at 30–90 days compared with BIVP (13.1% ± 10.8% vs. 10.1% ± 8.8%; β = −2.99; *p* = 0.030). After IPW‐PS adjustment, the difference remained significant (adjusted β = −2.61; *p* = 0.046), although the absolute magnitude—approximately a 3‐point lower mean ΔLVEF—was not clinically meaningful. Median LVEF increased from 30% [25–35] to 45% [35–50] with LBBAP, and from 30% [25–33] to 45% [35–52] with BIVP. At 1 year, LVEF improvement remained slightly lower in the LBBAP group (ΔLVEF = 12.6% ± 12.0% vs. 16.1% ± 12.1%; β = −3.51; *p* = 0.029), persisting after multivariable adjustment (adjusted β = −3.18; *p* = 0.036). The absolute difference, however, was modest and likely of limited clinical relevance. Median LVEF at 1 year was similar between groups (45% [35–50] vs. 45% [35–52]; *p* = 0.438).

**FIGURE 3:**
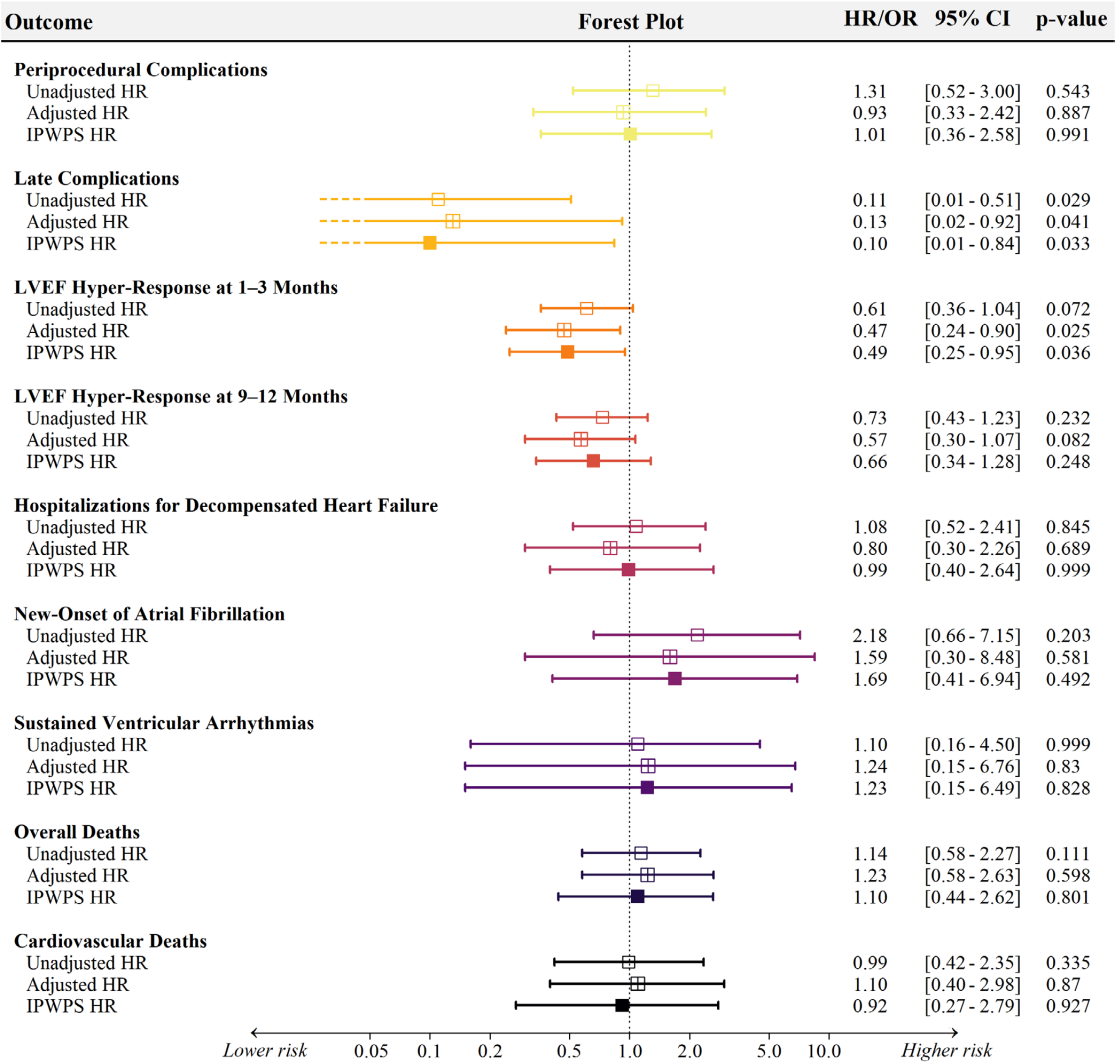
Crude and adjusted analyses of clinical outcomes associated with LBBAP compared with BIVP. Forest plot displaying unadjusted, covariate‐adjusted, and inverse probability–weighted (IPW‐PS) hazard ratios (HRs) or odds ratios (ORs) for major procedural and clinical outcomes. Horizontal bars represent 95% confidence intervals, with the dashed vertical line indicating a neutral effect (HR/OR = 1). BIVP = biventricular pacing; LBBAP = left bundle branch area pacing.

Early hyper‐response (≥ 15‐point LVEF increase) occurred in 38.7% of LBBAP patients versus 50.6% with BIVP (*p* = 0.072; OR 0.61, 95% CI 0.36–1.04), reaching statistical significance after IPW‐PS adjustment (OR 0.49, 95% CI 0.25–0.95; *p* = 0.036). At 9–12 months, hyper‐response rates remained comparable between groups (52.0% vs. 59.8%; *p* = 0.231; adjusted OR 0.66, 95% CI 0.34–1.28; *p* = 0.248) (Figures 2B and 3). Two patients in the LBBAP group with persistent LVEF non‐response were later upgraded to BIVP, both subsequently showing improvement in LVEF (+11% at a mean follow‐up of 6 months).

Hospitalization for heart failure occurred at comparable rates between groups (11.1 vs. 7.1 events per 100 patient‐years; *p* = 0.390). In both crude and IPW‐PS–adjusted analyses, LBBAP was not associated with an increased risk of heart‐failure hospitalization (crude HR 1.08, 95% CI 0.52–2.41; *p* = 0.845; adjusted HR 0.99, 95% CI 0.40–2.64; *p* > 0.999). The incidence of new‐onset AF was also similar (4.9 vs. 2.3 events per 100 patient‐years; HR 2.18, 95% CI 0.66–7.15; *p* = 0.203), a finding confirmed after IPW‐PS adjustment (HR 1.69, 95% CI 0.41–6.94; *p* = 0.492). Rates of sustained ventricular arrhythmias were low and comparable between groups (2.4 vs. 2.5 events per 100 patient‐years; crude HR 1.10, 95% CI 0.16–4.50; *p* > 0.999; adjusted HR 1.23, 95% CI 0.15–6.49; *p* = 0.828). Similarly, LBBAP was not associated with excess all‐cause mortality (crude HR 1.14, 95% CI 0.58–2.27; *p* = 0.672; adjusted HR 1.10, 95% CI 0.44–2.62; *p* = 0.801) or cardiovascular death (crude HR 0.99, 95% CI 0.42–2.35; *p* > 0.999; adjusted HR 0.92, 95% CI 0.27–2.79; *p* = 0.927). Detailed causes of death are summarized in Table 4 and Table S8.

**TABLE 4:** Cause of Deaths.

Cause of death^a^	LBBAP (*n* = 75)	BIVP (*n* = 239)	*p*‐value
Cardiovascular	8 (10.7%)	16 (6.7%)	0.259
Pump failure	5 (6.7%)	7 (2.9%)	0.160
Sudden death	0 (0%)	5 (2.1%)	0.320
Electrical storm	2 (2.7%)	2 (0.8%)	0.600
Other	1 (1.3%)	2 (0.8%)	0.600
Non‐cardiovascular	5 (6.7%)	9 (3.8%)	0.335
Cancer	3 (4.0%)	3 (1.3%)	0.150
Non‐cancer	1 (1.3%)	6 (2.5%)	0.680

^a^ Data are *n* (%).

#### 3.2.1 Supplementary Analyses

##### 3.2.1.1 Sensitivity Analysis

In a sensitivity analysis excluding rescue LBBAP cases after failed coronary sinus lead implantation and restricting the comparison to patients treated according to the initial successful implantation strategy, results were consistent with the main analysis. LBBAP remained associated with a lower risk of late complications (HR: 0.23; 95% CI: 0.03–0.99; *p* = 0.048), with no significant differences in periprocedural major complications (OR: 1.27; 95% CI: 0.41–3.66; *p* = 0.665), LVEF hyper‐response at 1 to 3 months (OR: 0.52; 95% CI: 0.25–1.06; *p* = 0.074) or 9 to 12 months (OR: 0.64; 95% CI: 0.31–1.37; *p* = 0.287), heart failure hospitalization (HR: 0.90; 95% CI: 0.32–2.85; *p* = 0.764), new‐onset AF (HR: 0.68; 95% CI: 0.16–2.92; *p* = 0.603), sustained ventricular arrhythmias (HR: 1.15; 95% CI: 0.05–8.39; *p* = 0.927), all‐cause mortality (HR: 1.19; 95% CI: 0.46–3.42; *p* = 0.541), or cardiovascular mortality (HR: 1.03; 95% CI: 0.24–2.91; *p* = 0.959).

##### 3.2.1.2 Device‐Related Events Not Meeting the Definition of Major Complication

In the LBBAP group, 6 patients (8.0%) developed loss of LBB capture without requiring reintervention; 4 events occurred before discharge and 2 during follow‐up. The 9‐ to 12‐month LVEF hyper‐response rate was numerically lower in these patients than in those with preserved LBB capture, but the difference was not significant (33.3% vs. 53.6%; *p* = 0.419). Only 1 patient had a pacing threshold > 2.0 V at 0.4 ms during follow‐up. In the BIVP group, 9 patients (3.8%) had an LV lead threshold > 2.0 V at 0.4 ms, and 3 (1.3%) had LV lead micro‐dislodgement not requiring reintervention.

## 4 Discussion

Our findings complement the growing body of evidence comparing conduction system pacing (CSP) with conventional CRT. In this observational cohort, LBBAP was associated with shorter paced QRS duration, shorter procedural time, and a comparable rate of periprocedural complications compared with BIVP. Device‐related complications during follow‐up were less frequent in the LBBAP group, with approximately 10‐fold fewer lead‐related complications. Reverse remodeling appeared broadly similar between groups, with comparable hyper‐response rates at 1 year. Likewise, major clinical outcomes—including heart‐failure hospitalization, new‐onset AF, sustained ventricular arrhythmias, and all‐cause mortality—did not differ significantly between LBBAP and BIVP, suggesting similar outcomes between strategies in this observational cohort.

Compared with our largely neutral findings regarding LVEF response, heart‐failure hospitalizations, and arrhythmic outcomes, several prior studies have suggested potential advantages of LBBAP over BIVP. In the LBBP‐RESYNC trial [10], 40 nonischemic LBBB (Left bundle branch block) patients randomized to LBBAP or BIVP showed greater 6‐month LVEF gain with LBBAP (+ 13% vs. + 7%; *p* = 0.039) and larger reductions in LV volumes and NT‐proBNP. However, the modest LVEF increase in the BIVP arm—lower than in larger CRT trials—suggests caution in extrapolating small‐sample results [10]. In a large multicenter study of 1778 patients, Vijayaraman et al. (2023) reported greater LVEF improvement with LBBAP (27% → 41% vs. 27% → 37%; *p* < 0.001) and a lower 2‐year composite of death or heart‐failure hospitalization (20.8% vs. 28%; HR 0.67), though the absolute effect size was modest (~4%) [12]. Similarly, in a prospective multicenter study of 415 CRT candidates, Diaz et al. (2024, JACC EP) found that LBBP achieved higher freedom from the composite of HF hospitalization and all‐cause mortality compared with BIVP (HR 1.43; 95% CI 1.18–1.73; *p* < 0.001) [14]. Chen et al. (2021) also demonstrated that LBBAP‐CRT achieved greater QRS narrowing and larger LVEF increases than optimized adaptive BIVP (ΔLVEF + 21% vs. + 15%; *p* = 0.015), with similar rates of clinical events [13]. Smaller controlled studies, such as Wang et al. (2020), corroborated these findings, reporting higher CRT response rates with LBBAP (100% vs. 63%; *p* = 0.038) [18]. Finally, an updated meta‐analysis by Lakshman et al. (2024) pooling randomized and observational data reported that LBBAP was associated with greater reductions in LV volumes, shorter QRS duration, improved NYHA class, and fewer HF hospitalizations compared with BIVP, while maintaining similar safety [19]. Regarding arrhythmias, the only study specifically addressing this question—by Herweg et al. (2024)—reported that LBBAP was associated with a significantly lower incidence of both sustained VT/VF (HR 0.46) and new‐onset AF (HR 0.34) [20].

On the other hand, the LEVEL‐AT trial (Pujol‐Lopez et al. JACC EP *2022*) randomized 70 CRT candidates and demonstrated that conduction system pacing was noninferior to BIVP in shortening LV activation time, reducing LV volumes, and preventing 6‐month HF hospitalization or death (2.9% vs. 11.4%; *p* = 0.002 for noninferiority). These findings align with our results showing broadly similar reverse remodeling across pacing modalities [21] More recently, the CSPOT study (Jastrzębski et al. Circulation EP 2024) provided acute hemodynamic evidence comparing LBBAP, BIVP, and combined “left bundle‐optimized” CRT (LOT‐CRT). LOT‐CRT and BIVP achieved greater increases in LV dP/dt_max_ than isolated LBBAP, particularly in patients with very wide QRS or deep septal capture, suggesting that the addition of a coronary‐vein LV lead may further optimize resynchronization in selected cases [22].

Taken together, our study contributes to the ongoing debate regarding the place that would take LBBAP in CRT indications. Our findings should not be interpreted as contradicting prior studies that suggested advantages of LBBAP, but rather as indicating that such advantages may not be consistently demonstrable across all settings. In particular, in an older and more comorbid cohort, and inclusion of rescue LBBAP cases after failed coronary sinus lead implantation, LBBAP was associated with similar echocardiographic and clinical outcomes to BIVP, together with fewer late device‐related complications. These results therefore support a more nuanced interpretation of the literature, in which the relative performance of LBBAP and BIVP may depend on patient profile and procedural context.

These findings should be interpreted with several limitations. The retrospective, nonrandomized design introduces selection bias, as treatment choice may have been influenced by patient characteristics. Despite extensive propensity score adjustment, residual confounding cannot be excluded. LBBAP was more often used in older or previously failed CRT patients, which could have attenuated its apparent benefit. Importantly, follow‐up was substantially shorter in the LBBAP group because of its later adoption, which may have limited the detection of later clinical and device‐related events despite the use of time‐to‐event analyses accounting for unequal observation time. Late complication rates should also be interpreted cautiously, as complication profiles differ between BIVP and LBBAP, and the shorter follow‐up in the LBBAP group may have limited detection of later LBBAP‐specific events, particularly loss of conduction capture. Arrhythmic outcomes should likewise be considered exploratory, as new‐onset AF was identified using routine ECG and noncontinuous Holter monitoring, which may have underestimated asymptomatic or intermittent episodes, and the imbalance in CRT‐D use between groups may have influenced ascertainment of sustained ventricular arrhythmias. The limited sample size for LBBAP reduced power for rare endpoints such as new‐onset AF or VT/VF. Echocardiographic measures were obtained in routine practice rather than core‐lab adjudicated, introducing possible variability, though use of broad response categories likely mitigated this. Finally, we did not distinguish selective from non‐selective LBBAP capture, which may influence outcomes.

## 5 Conclusion

Our findings support the idea that left bundle branch area pacing (LBBAP) should no longer be viewed solely as a rescue option after failed coronary sinus lead placement but rather as a promising and physiologic alternative to conventional biventricular pacing for cardiac resynchronization. In our real‐world experience, LBBAP achieved comparable improvements in ventricular function and clinical outcomes while being associated with fewer device‐related complications, suggesting that LBBAP may represent an alternative CRT strategy in selected patients. Although our study was not powered to detect small differences in mortality or arrhythmic events, the procedural and safety findings observed with LBBAP may be clinically meaningful, particularly in elderly or frail patients. These results should be confirmed in larger prospective randomized studies before definitive conclusions can be drawn regarding the comparative role of LBBAP and BIVP in de novo CRT implantation.

## Funding

This study was completely academic without any funding, conducted by the clinic investigation center of the university hospital of Brest. Fawzi Kerkouri, Laurent Palud, Jean Baptiste Moreau, Vincent Mansourati, Hugo Hager, Pierre Khattar, Valérie Valls‐Bertault, Jean Philippe Hacot, and Florent Le Ven have no relevant conflicts of interest. Jacques Mansourati is consultants and/or received fees from Medtronic, Abbott, Boston scientific, Biotronik and Microport.

## Ethics Statement

Informed consent was obtained for all participants, and the study adhered to the principles outlined in the Helsinki Declaration. The institutional review board and the French Data Protection Committee approved the retrospective analysis of de‐identified clinical data.

## Conflicts of Interest

The authors declare no conflicts of interest.

## Supporting information


**Table S1:** Recorded baseline characteristics.
**Table S2:** Outcome events.
**Table S3:** Included Variables in adjusted models.
**Figure S1:** Loveplot before and after IPW PS.
**Table S4:** Procedural data and acute electrical parameters.
**Table S5:** Other major complications.
**Table S6:** Periprocedural Events not fulfilling major complication definition.
**Table S7:** Early and Late Reoperations.
**Table S8:** Causes of deathss.

## Data Availability

The data that support the findings of this study are available on request from the corresponding author. The data are not publicly available due to privacy or ethical restrictions.
