# Supplementary material for: Left Bundle Branch Area Pacing vs. Biventricular Pacing for Cardiac Resynchronization: Propensity Score Analysis
Source: J Arrhythm. 2026 May 6;42(3):e70359. doi: 10.1002/joa3.70359 (PMC13149756; doi:10.1002/joa3.70359)
Supplement: Supplementary file 1 — Table S1: Recorded baseline characteristics. Table S2: Outcome events. Table S3: Included Variables in adjusted models. Figure S1: Loveplot before and after IPW PS. Table S4: Procedural data and acute electrical parameters. Table S5: Other major complications. Table S6: Periprocedural Events not fulfilling major complication definition. Table S7: Early and Late Reoperations. Table S8: Causes of deathss. [file JOA3-42-e70359-s001.docx]

**Left Bundle Branch Area Pacing vs. Biventricular Pacing for Cardiac Resynchronization: Propensity Score Analysis**

**Supplementary Appendix**

**Table of Contents**

Supplementary Table 1. Recorded baseline characteristics…………………………………..2

Supplementary Table 2. Outcome events……………………………………………………..3

Supplementary Table 3. Included Variables in adjusted models……………………………...4

Supplementary Figure 1. Loveplot before and after IPW PS………………………………….4

Supplementary Table 4. Procedural data and acute electrical parameters……………………..5

Supplementary Table 5. Other major complications…………………………………………...6

Supplementary Table 6. Periprocedural Events not fulfilling major complication definition…6

Supplementary Table 7. Early and Late Reoperations…………………………………………6

Supplementary Table 8. Causes of deaths………………………………………………………..7

**Supplementary** **Table 1. Recorded baseline characteristics**

|  | Measured Characteristics |
| --- | --- |
| Demographic Data at the Implant Procedure | 1. Age, Sex, Hight & Weight 2. Heart disease: ischemic, dilated cardiomyopathy, other with details 3. Comorbidities:  - Diabetes: All types of diabetes were considered - Hypertension; hypercholesterolemia - Congestive heart failure: with NYHA class - Coronary artery disease - Atrial fibrillation: Paroxysmal or persistent/permanent - Severe respiratory disease: Chronic respiratory failure under oxygen, COPD with MEVS < 50 ml/min or restrictive respiratory failure with TLC < 50% - Severe psychiatric troubles: psychotic, depression or bipolar disorders with ongoing treatment. - Dementia: moderate/severe dementia with MMSE score < 20 - Dialysis; severe chronic kidney disease: GFR < 30 ml/min - Cirrhosis - Cancer; Malignant hemopathy: active or in remission - Severe impaired mobility: difficulty to manage daily activities with need of some assistance from others. - Transaortic valve replacement (TAVR)  1. Type of Pacing: LBBAP or BVP 2. ECG at implant:   - Rhythm at implant: Sinus rhythm, atrial fibrillation, PR >200s  - QRS duration (ms) and QRS morphology:  a) Typic LBBB: QRS ≥130ms; broad, notched R waves and  absence q waves in lateral leads, QS or rS in V1–V3  b) Non-typic LBBB  c) RBBB  d) Paced QRS   1. Imaging data:   - Left ventricular ejection fraction (LVEF) in % measured by transthoracic  echocardiography  - Left ventricular end-diastolic volume in ml/m²  - Right ventricular dysfunction: presence and severity  - Performed Cardiac MRI: yes or no; Late gadolinium  enhancement: yes or no, number of segments and localization   1. History of pacemaker:  - Type of previous pacemaker: transvenous, epicardial or leadless pacemaker - Reason for switching to CRT: device infection, lead dysfunction, pacing induced cardiomyopathy, LVEF decline, other reason with details  1. Treatment: Valsartan/Neprilysin, Beta-blocker, SGLT2 inhibitor, Aldosterone antagonist, Furosemide, ACEi/ARB, Anticoagulant |
| Procedural Data | 1. Implant date; device, leads and sheath brands, venous access 2. Pacing threshold (V/ms), impedance (Ω), ventricular detection (mV) 3. Procedure and fluoroscopy duration 4. Associated implantable cardioverter defibrillator (ICD) 5. Electrophysiologic data post implant:   - QRS morphology: Qr, qR, rSr’ or rSR’ wave in V1  - QRS duration (ms)  - LVAT, V1-V6 for LBBAP |

**Supplementary** **Table 2. Outcome events**

|  | Measured events |
| --- | --- |
| Complications Prior to Discharge | 1. Nature of complication: Pericardial effusion, lead micro dislodgement, lead macro dislodgement, lead impingement, tricuspid Injury, vascular complication, pneumothorax, loss of LBB capture (Loss of right ventricular pre-excitation after implant; LVAT rise after implant; V1-V6 decrease after implant; QRS duration rise after implant), threshold rise (>3V) after implant, coronary sinus dissection, septal perforation, pocket hematoma, infection, death related to device/implantation, other with details 2. Severity: minor or Major complication: adverse event resulting in death, invasive intervention or prolonged hospitalization (>48h) 3. Modality of management: wait & see, percutaneous drainage, pocket revision, lead repositioning, device extraction (with/without new implant, type of new device), cardiac surgery, non-cardiac surgery, other intervention with details. |
| Complications During Follow-up | 1. Nature of complication: local (hematoma, local infection, systemic infection, erosion, generator migration), Lead related (fracture, macro-dislodgement, micro-dislodgement, threshold rise>3V/0.4ms, detection troubleshooting), loss of LBB capture, vascular occlusion, chronic pain/discomfort, other with details 2. Severity: minor or Major complication: adverse event resulting in death, invasive intervention or rehospitalization. 3. Modality of management: wait & see, percutaneous drainage, pocket revision, lead repositioning, device extraction (with/without new implant, type of new device), cardiac surgery, non-cardiac surgery, other intervention with details. 4. Date of complication |
| Pacemaker and LVEF data | 1. ventricular pacing %, threshold, impedance, detection, date of recording 2. Premature ventricular complexes burden >10% 3. All LVEF evaluations with dates. 4. Switch LBBAP to BVP/BVP to LBBAP, date of switch |
| Clinical data | 1. Hospitalization for heart failure decompensation: any hospital admission for decompensated heart failure (worsening symptoms with objective signs such as pulmonary edema requiring intravenous therapy). Proportion of patients with ≥1 hospitalization, date and number of hospitalizations. 2. Onset of atrial fibrillation: paroxysmal, persistent or permanent 3. Sustained ventricular arrythmias (VT/VF) 4. NYHA Class |
| Death | 1. Cause of death:   - Cardiovascular: with details  - Non-cardiovascular: with details  - Device-related: with details about the circumstances of death  - Unknown origin |

**Supplementary Table 3. Included Variables in adjusted models**

| **Early Complications** | Age, Sex, BMI, Heart disease, LVEF, Diabetes, Severe respiratory disease, Cancer, Anticoagulants |
| --- | --- |
| **Late Complications** | Age, Sex, BMI, Heart disease, LVEF, Diabetes, Severe respiratory disease, Dialysis, Cancer, Severe impaired mobility, Pacemaker history, Anticoagulants |
| **LVEF hyper-response at 1–3 months** | Age, Sex, BMI, Heart disease, Rhythm at implant, QRS duration, QRS morphology, LVEF, LVEDV, Number of segments of Late gadolinium enhancement, NYHA class, Diabetes, Atrial fibrillation, Dialysis, Valsartan/Neprilysin, ACEi/ARB, Beta-blocker, SGLT2 inhibitor, Aldosterone antagonist |
| **LVEF hyper-response at 9–12 months** | Age, Sex, BMI, Heart disease, Rhythm at implant, QRS duration, QRS morphology, LVEF, LVEDV, Number of segments of Late gadolinium enhancement, NYHA class, Diabetes, Atrial fibrillation, Dialysis, Valsartan/Neprilysin, ACEi/ARB, Beta-blocker, SGLT2 inhibitor, Aldosterone antagonist |
| **Hospitalization for heart failure decompensation** | Age, Sex, BMI, Heart disease, LVEF, NYHA class, Diabetes, Atrial fibrillation, Dialysis, Valsartan/Neprilysin, ACEi/ARB, Beta-blocker, SGLT2 inhibitor, Aldosterone antagonist |
| **New onset atrial fibrillation** | Age, Sex, BMI, Heart disease, LVEF, Hypertension, Dialysis, Severe respiratory disease, Beta-blocker |
| **Sustained ventricular arrythmias** | Age, Sex, BMI, Heart disease, LVEF, Late gadolinium enhancement, Coronary artery disease, Beta-blocker |
| **Overall deaths** | Age, Sex, BMI, Heart disease, LVEF, NYHA class, Diabetes, Severe respiratory disease, Dialysis, Severe impaired mobility, Cirrhosis,  Cancer, Associated ICD, Valsartan/Neprilysin, SGLT2 inhibitor |
| **Cardiovascular deaths** | Age, Sex, BMI, Heart disease, LVEF, NYHA class, Diabetes, Dialysis, Associated ICD, Valsartan/Neprilysin, SGLT2 inhibitor |

**Supplementary Figure 1. Loveplot before and after IPW PS**

**
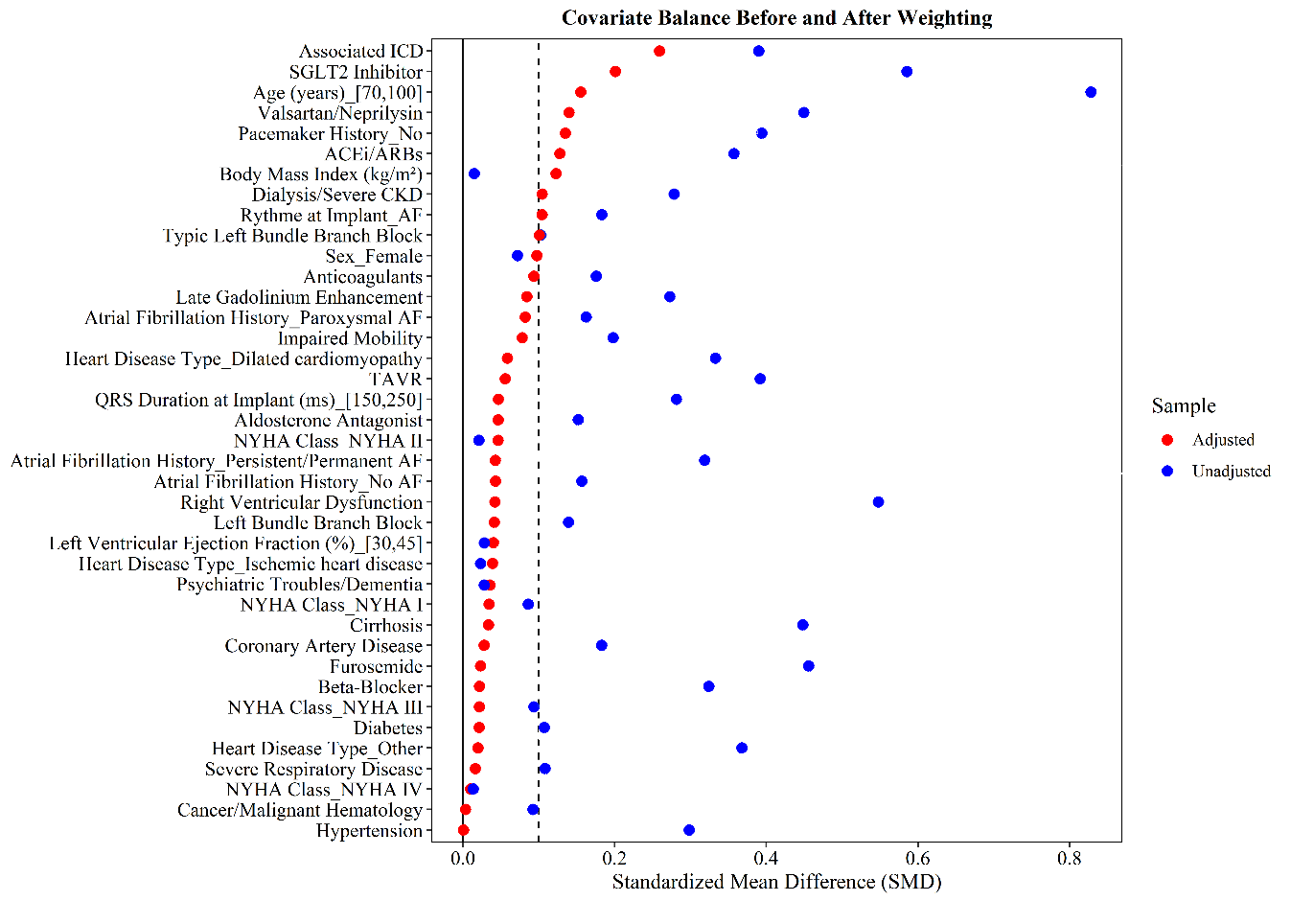
**

**Supplementary Table 4. Procedural data and acute electrical parameters**

| **Characteristic*** | **LBBAP** N = 75 | **BIVP** N = 239 | **p-value** |
| --- | --- | --- | --- |
| **Procedural time** | 96 [58 – 121] | 128 [100 – 157] | < 0.001 |
| **Fluoroscopy time** | 18 [12 – 34] | 23 [16 – 37] | 0.018 |
| **Venous Access** |  |  | 0.442 |
| *Subclavian vein* | 68 (90.7%) | 214 (89.5%) |  |
| *Axillary vein* | 1 (1.3%) | 4 (1.7%) |  |
| *Cephalic and subclavian vein* | 6 (8.0%) | 21 (8.8%) |  |
| **Device manufacturer** |  |  | <0.001 |
| *Medtronic* | 11 (14.7%) | 28 (11.7%) |  |
| *Abbott* | 37 (49.3%) | 58 (24.3%) |  |
| *Boston scientific* | 6 (8.00%) | 45 (18.8%) |  |
| *Biotronik* | 17 (22.7%) | 80 (33.5%) |  |
| *Microport* | 4 (5.33%) | 28 (11.7%) |  |
| **Lead (for LBBAP/CRT)** |  |  | <0.001 |
| *Medtronic* | 32 (42.7%) | 25 (10.5%) |  |
| *Abbott* | 36 (48.0%) | 92 (38.5%) |  |
| *Boston scientific* | 1 (1.33%) | 30 (12.6%) |  |
| *Biotronik* | 6 (8.00%) | 81 (33.9%) |  |
| *Microport* | 0 (0%) | 11 (4.60%) |  |
| **Sheath manufacturer** |  |  | <0.001 |
| *Medtronic* | 30 (40.0%) | 23 (9.62%) |  |
| *Abbott* | 38 (50.6%) | 87 (36.4%) |  |
| *Boston scientific* | 0 (0%) | 40 (16.7%) |  |
| *Biotronik* | 7 (9.33%) | 78 (32.6%) |  |
| *Microport* | 0 (0%) | 11 (4.60%) |  |
| **Pacing threshold—V** | 0.9 [0.6 - 1.4] | 1.0 [0.7 - 1.4] | 0.460 |
| **Pacing impedance—Ω** | 669 [540 - 807] | 852 [630 - 1,080] | <0.001 |
| **Detection—mV** | 7.4 [5.0 -12.0] | 10.0 [6.7 - 15.0] | 0.005 |
| **QRS Width**^†^**—ms** | 128 [119 - 139] | 139 [127 - 148] | <0.001 |
| **Qr, qR, rSr’ or rSR’ wave in V1** | 65 (86.7%) | 225 (94.1%) | 0.034 |
| **Stim–LVAT—ms** | 75 [69 - 85] | **—** |  |
| **V1–V6 interpeak interval—ms** | 40 [35 - 45] | **—** |  |
| **Associated ICD** | 22 (29.3%) | 132 (55.2%) | <0.001 |

*Data are mean ± sd or n (%). ^†^Missing data: 4 for LBBA Pacing, 5 for CRT.

**Supplementary Table 5. Other major complications**

| **Characteristic*** | **LBBAP** N = 75 | **BIVP** N = 239 | **p-value** |
| --- | --- | --- | --- |
| **Other early major complications** | **2 (2.67%)** | **1 (0.42%)** | **0.143** |
|  |  |  | >0.999 |
| Threshold elevation requiring reoperation | 1 (50.0%) | **—** |  |
| Phrenic stimulation requiring reoperation | **—** | 1 (100.0%) |  |
| Stroke post-anticoagulant discontinuation | 1 (50.0%) | **—** |  |
| **Other late major complications** | **—** | 5 (2.09%) | 0.343 |
|  |  |  | >0.999 |
| Diaphragmatic contraction requiring reoperation | **—** | 4 (80.0%) |  |
| Threshold rise requiring reoperation | **—** | 1 (20.0%) |  |

**Supplementary Table 6. Periprocedural Events not fulfilling major complication definition**

| **Characteristic*** | **LBBA Pacing** N = 75 | **CRT** N = 239 | **p-value** |
| --- | --- | --- | --- |
| **Loss of LBB capture** | 4 (5.33%) | 0 (0%) | 0.003 |
| *Loss r' after implant* | 4 (5.33%) | 0 (0%) | 0.003 |
| *LVAT rise after implant* | 1 (1.33%) | 0 (0%) | 0.239 |
| *V1–V6 decrease after implant* | 1 (1.33%) | 0 (0%) | 0.239 |
| *QRS duration rise after implant* | 1 (1.33%) | 0 (0%) | 0.239 |
| **Threshold rise at J1 post-implant** | 0 (0%) | 1 (0.42%) | >0.999 |

**Supplementary Table 7. Early and Late Reoperations**

| **Characteristic*** | **LBBAP** N = 75 | **BIVP** N = 239 | **p-value** |
| --- | --- | --- | --- |
| **Pre-discharge reoperations** | **5 (6.7%)** | **9 (3.8%)** | **0.290** |
| Pericardial effusion | 0 (0%) | 1 (0.4%) | >0.999 |
| Lead macro-dislodgement | 0 (0%) | 4 (1.7%) | 0.576 |
| Loss of LBB capture | 1 (1.3%) | 0 (0%) | 0.239 |
| Pneumothorax | 2 (2.7%) | 3 (1.3%) | 0.596 |
| Death related to implantation | 1 (1.3%) | 0 (0%) | 0.239 |
| Other | 2 (2.7%) | 1 (0.4%) | 0.143 |
|  |  |  | >0.999 |
| *- Threshold elevation requiring reoperation* | 1 (50.0%) | **—** |  |
| *- Phrenic stimulation requiring reoperation* | **—** | 1 (100.0%) |  |
| *- Stroke post-anticoagulant discontinuation* | 1 (50.0%) | **—** |  |
| **Late reoperations** | **1 (1.3%)** | **19 (7.9%)** | **0.036** |
| Infection | 0 (0%) | 6 (2.5%) | 0.342 |
| Lead dislodgment | 0 (0%) | 8 (3.4%) | 0.016 |
| Loss of LBB capture requiring reoperation | 1 (1.3%) | 0 (0%) | 0.239 |
| Other | 0 (0%) | 5 (2.1%) | 0.343 |
|  |  |  | >0.999 |
| *- Diaphragmatic contraction requiring reoperation* | — | 4 (80.0%) |  |
| *- Threshold rise requiring reoperation* | — | 1 (20.0%) |  |

*Data are n (%).

**Supplementary Table 8. Causes of deaths**

| **Characteristic** | **LBBA Pacing** N = 75 | **CRT** N = 239 | **p-value** |
| --- | --- | --- | --- |
| **Cause of death** |  |  | >0.999 |
| *Cardiovascular* | 8 (61.5%) | 16 (64.0%) |  |
| *Non-Cardiovascular* | 5 (38.5%) | 9 (36.0%) |  |
| **Cardiovascular causes** |  |  | 0.176 |
| *Acute coronary syndrome* | 0 (0%) | 2 (12.5%) |  |
| *Electrical storm* | 2 (25.0%) | 2 (12.5%) |  |
| *Pomp failure* | 5 (62.5%) | 7 (43.8%) |  |
| *Pulmonary Hypertension* | 1 (12.5%) | 0 (0%) |  |
| *Sudden cardiac death* | 0 (0%) | 5 (31.3%) |  |
| **Non-Cardiovascular causes** |  |  | 0.217 |
| *Anticoagulant-related bleeding* | 0 (0%) | 2 (22.2%) |  |
| *Colorectal cancer* | 0 (0%) | 1 (11.1%) |  |
| *Liver cancer* | 0 (0%) | 1 (11.1%) |  |
| *Lung cancer* | 1 (25.0%) | 1 (11.1%) |  |
| *Autoimmune encephalitis* | 0 (0%) | 2 (22.2%) |  |
| *Malignant hemopathy* | 2 (50.0%) | 0 (0%) |  |
| *Road traffic accident* | 0 (0%) | 2 (22.2%) |  |
| *Chronic respiratory failure* | 1 (25.0%) | 0 (0%) |  |
